# Activation and sensitization of meningeal nociceptors by PACAP-38: implications for migraine headache

**DOI:** 10.1093/brain/awaf284

**Published:** 2025-08-06

**Authors:** Rune Häckert Christensen, Andrew Strassman, Messoud Ashina, Håkan Ashina, Rami Burstein

**Affiliations:** Department of Neurology, Danish Headache Center, Copenhagen University Hospital - Rigshospitalet, Copenhagen 2100, Denmark; Department of Clinical Medicine, Faculty of Health and Medical Sciences, University of Copenhagen, Copenhagen 2200, Denmark; Harvard Medical School, Boston, MA 02115, USA; Department of Anesthesia, Critical Care and Pain Medicine, Beth Israel Deaconess Medical Center, Boston, MA 02215, USA; Harvard Medical School, Boston, MA 02115, USA; Department of Anesthesia, Critical Care and Pain Medicine, Beth Israel Deaconess Medical Center, Boston, MA 02215, USA; Department of Neurology, Danish Headache Center, Copenhagen University Hospital - Rigshospitalet, Copenhagen 2100, Denmark; Department of Clinical Medicine, Faculty of Health and Medical Sciences, University of Copenhagen, Copenhagen 2200, Denmark; Department of Neurology, Danish Headache Center, Copenhagen University Hospital - Rigshospitalet, Copenhagen 2100, Denmark; Department of Clinical Medicine, Faculty of Health and Medical Sciences, University of Copenhagen, Copenhagen 2200, Denmark; Harvard Medical School, Boston, MA 02115, USA; Department of Anesthesia, Critical Care and Pain Medicine, Beth Israel Deaconess Medical Center, Boston, MA 02215, USA; Harvard Medical School, Boston, MA 02115, USA; Department of Anesthesia, Critical Care and Pain Medicine, Beth Israel Deaconess Medical Center, Boston, MA 02215, USA

**Keywords:** migraine, electrophysiology, post-traumatic headache, headache, craniotomy, meningeal nociceptors

## Abstract

Emerging evidence implicates pituitary adenylate cyclase-activating polypeptide-38 (PACAP-38) in migraine and other headache disorders. Systemic infusion of PACAP-38 induces migraine headache in people with migraine and mild headache in healthy adults. However, the precise mechanism and site of action remain poorly characterized. To address this gap, we assessed whether PACAP-38 elicits headache by activating meningeal nociceptors.

*In vivo* single-unit electrophysiological recordings were obtained from C- and Aδ-meningeal nociceptors in the trigeminal ganglion of anaesthetized male and female rats. Spontaneous firing rates and mechanical responses to dural indentation with von Frey filaments were recorded before and for 4 h after a 20-min intracarotid infusion of PACAP-38 (1 µg/ml/kg, 10 µg/ml/kg and 100 µg/ml/kg) or vehicle (isotonic saline).

At the human-equivalent dose (10 µg/ml/kg), PACAP-38 activated 15 of 30 (50%) neurons, compared with none after vehicle (*P* < 0.01). Activation rates did not differ between C-fibres (7 of 17; 41%) and Aδ-fibres (8 of 13; 61.5%). The lowest dose of PACAP-38 (1 µg/ml/kg) activated 3 of 9 (33%) neurons, whereas the highest dose (100 µg/ml/kg) activated all four tested neurons, including two C-fibres and two Aδ-fibres.

These results suggest that PACAP-38 induces headache through peripheral activation of meningeal nociceptors rather than exerting direct central effects within the spinal trigeminal nucleus.

## Teaser

Using *in vivo* single-unit electrophysiological recordings in rats, Christensen *et al.* show that infusion of PACAP-38 activates and sensitizes meningeal nociceptors in the trigeminal ganglion. This likely accounts for the role of PACAP-38 in the development of migraine headache, with implications for future treatments.

## Introduction

Headache disorders, including migraine, impose an enormous socioeconomic burden on global health.^1^ They manifest as recurring, disabling attacks of moderate to severe headache, accompanied by photophobia, phonophobia, nausea and vomiting.^2^ Their pathogenesis remains incompletely understood but is widely accepted to involve the trigeminovascular system, which includes trigeminal afferent projections to the pain-sensitive meninges and its blood vessels, referred to as meningeal nociceptors.^3,4^ Upon activation, these nociceptors send signals to the CNS via central components of the trigeminovascular pathway that culminate in the perception of headache.^3,4^ The identification of mediators that engage meningeal nociceptors is thus pivotal for mapping the neurobiological basis of headache disorders and guiding the development of targeted therapies.

One promising mediator is pituitary adenylate cyclase-activating polypeptide-38 (PACAP-38), a multifunctional peptide implicated in processes related to vascular tone, nociception and immune responses.^5^ PACAP-38 is found in the dura where it is thought to be released from sensory and parasympathetic nerve fibres.^6-9^ Its G protein-coupled receptors are expressed on sensory nerve fibres, vascular smooth muscle cells (VSMCs) and mast cells.^8,10-13^ In experimental studies, systemic PACAP infusion induces migraine headache in people with migraine, whereas healthy adults experience only a mild headache.^14^ However, the mechanism and site of action underlying PACAP-induced headache have yet to be clarified.

We hypothesize that PACAP-38 induces headache by activating and sensitizing dural nerve endings of meningeal nociceptors, whose cell bodies reside in the trigeminal ganglion. Our hypothesis aligns well with the results from a recent phase II trial,^15^ in which a monoclonal antibody—too large to cross the blood–brain barrier (BBB)—targeting the PACAP ligand significantly reduced the monthly number of days with migraine headache. These findings suggest that peripheral blockade of PACAP might suffice to disrupt the development of migraine headache.

In the present study, we investigated whether systemic infusion of PACAP-38 activates and/or sensitizes mechanosensitive meningeal nociceptors in both unmyelinated C-fibres and thinly myelinated Aδ-fibres. To ensure clinical relevance, we adopted a dosing regimen and infusion duration that closely mirror the protocols used to induce migraine headache in people with migraine.

## Materials and methods

### Study design

The primary goals of the current study were to assess whether PACAP-38 activated or sensitized meningeal nociceptors in rats. To investigate this, we performed 5-h electrophysiological single-unit recordings (including 1 h of baseline, and 4 h of recording after start of PACAP-38 infusion), after three doses of PACAP-38 as well as vehicle. In a subset of activated neurons, we likewise explored the effect of dural lidocaine on the spontaneous firing rate of the meningeal nociceptors.

Experiments were approved by the Beth Israel Deaconess Medical Center and Harvard Medical School animal care committees and were conducted according to the US National Institutes of Health Guide for the Care and Use of Laboratory Animals.

### Surgical preparation

**Figure 1:**
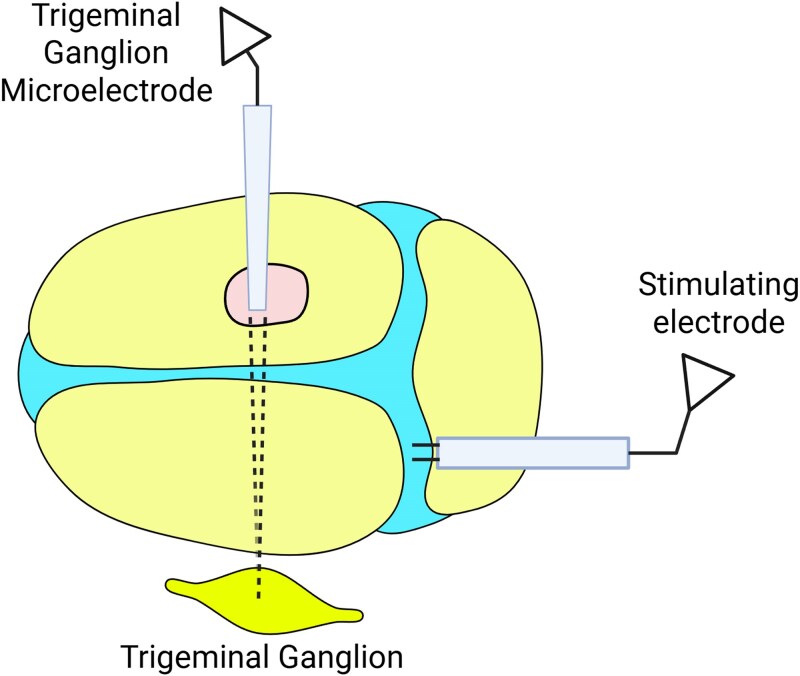
**Experimental set-up.** Created in BioRender. Häckert Christensen, R. (2025) https://BioRender.com/ujlxojs.

Male and female Sprague-Dawley rats (205–350 g) (Taconic) were anaesthetized with urethane (1.5 g/kg i.p.), treated with atropine (0.4 ml i.p.), and paralysed with rocuronium bromide (10 mg/ml, 1 ml/h continuous intravenous infusion). The femoral vein and carotid artery were cannulated for drug infusion (method developed by Agustin Melo-Carrillo at the Burstein Lab), and an endotracheal tube was placed to allow artificial ventilation with O_2_ inhalation. Core body temperature was maintained at 37°C using a feedback-controlled heating pad, and pO_2_ was maintained at >98% throughout the experiment. Once stabilized, rats were craniotomized on the left side to allow access for electrical and mechanical stimulation of the dura above the left transverse sinus, and on the right side (1–4 mm caudal to ∼1–4 mm lateral to the bregma) to allow an angled approach to the left trigeminal ganglion. The exposed dura was kept moist using isotonic saline. The experimental setup and orientation of electrodes is illustrated in Fig. 1.

### Neural recording

For single-unit recording from neurons in the trigeminal ganglion we used platinum-coated tungsten microelectrodes (impedance 150–300 kΩ). We investigated only a single neuron in each animal. Dural afferents were identified through their constant latency after electrical stimulation of the dura with a bipolar electrode (0.5 ms pulse, 5 mA, 0.75 Hz). Based on the latency and a 12 mm conduction distance to the trigeminal ganglion, neurons were classified as either Aδ (2–8 ms latency) or C-fibres (>8 ms latency). We used Spike2 software (CED, Cambridge, UK) for spike acquisition and waveform discrimination, including off-line analysis, to capture and analyse neuronal responses to PACAP-38 and mechanical stimulation of the dura. Mechanoreceptive fields of dural afferents were identified by probing the dura with blunt forceps or von Frey hairs. Based on previous experience, we studied mechanosensitive neurons with spontaneous activity above 0 spikes/min.

### Experimental paradigm

Spontaneous discharge activity was recorded continuously throughout the experiment (usually 5 h). After a baseline recording of 1 h, each animal received an infusion of either a single concentration of PACAP-38 (Sigma-Aldrich) or isotonic saline for 20 min through the common carotid artery. We opted for this route of PACAP-38 infusion, since measurements of neuropeptides released during migraine have so far most aptly been taken from the vasculature of the neck and cervical region and because intracarotid infusion permits more direct delivery to cephalic sites considered relevant to migraine and headache pathophysiology. PACAP-38 was administered at 1 µg/kg, 10 µg/kg or 100 µg/kg, dissolved in saline. All doses of PACAP-38, as well as isotonic saline, were administered at 1 ml/kg. Spontaneous activity was then recorded for up to 4 h. For a subset of neurons, lidocaine (2%) was applied topically to the dura to determine the contribution of dural receptive fields to the overall activity of the neurons in the trigeminal ganglion.

In a subset of neurons, we examined mechanical sensitivity by stimulating the dura perpendicularly with von Frey filaments within a range of forces. We used this to determine both (i) the lowest force needed for mechanical activation of a neuron (mechanical threshold) and (ii) the magnitude of the change in mechanical threshold.

Finally, for the main dose of PACAP-38 (10 µg/kg) and vehicle, we recorded heart rate immediately prior to infusion (baseline) and at 10-min intervals for 60 min after infusion using a rat pulse oximeter (Physiosuite^®^, Kent Scientific).

### Data analysis

For data analysis, we compared the mean neuronal firing during the 30 min baseline period that preceded the PACAP-38 infusion to their hourly firing rate after the infusion. A neuron was considered activated if the firing rate was 2 standard deviations (SD) above the mean of the baseline for at least 10 min (calculated in 60-s bins). The duration of activation was the number of 60-s bins above the mean + 2 SD. Response latency was the time from PACAP-38/placebo infusion to onset of activation. A neuron was considered sensitized if its mechanical threshold decreased from the threshold measured at baseline. No data points were excluded.

We performed sample size calculation for the human equivalent dose based on an anticipated equal likelihood of investigating an Aδ- or C-fibre. Based on an anticipated 40% difference in activation rate, 80% power and 5% alpha, one-sided, a group size of 15 in each group was estimated. For comparison with vehicle, an unequal group size was estimated using the same parameters, which estimated the vehicle group size as 10.

The proportion of activated neurons was compared between the PACAP-38 doses and vehicle using Fisher’s exact test. In addition, the effect of sex (male/female) and fibre type (Aδ/C-fibre) was examined using Fisher’s exact test and binomial logistic regression. The time to activation and magnitude of activation were compared between groups using the Wilcoxon Rank Sum test or Student’s *t*-test, depending on the normality of the data. Normality of data was tested using the Shapiro-Wilk’s test. We used Friedman’s test and Wilcoxon Signed Rank tests to compare firing rates before and after PACAP-38/vehicle. For this purpose, mean firing rates were compared between baseline and the 1st, 2nd, 3rd and 4th hour following PACAP-38/vehicle administration. We accepted statistical significance at *P* < 0.05. No corrections were performed for multiple comparisons. We performed all analyses in R version 4.2.0, using the ‘pwr’ package for sample size calculation.

## Results

### Neurons

**Figure 2:**
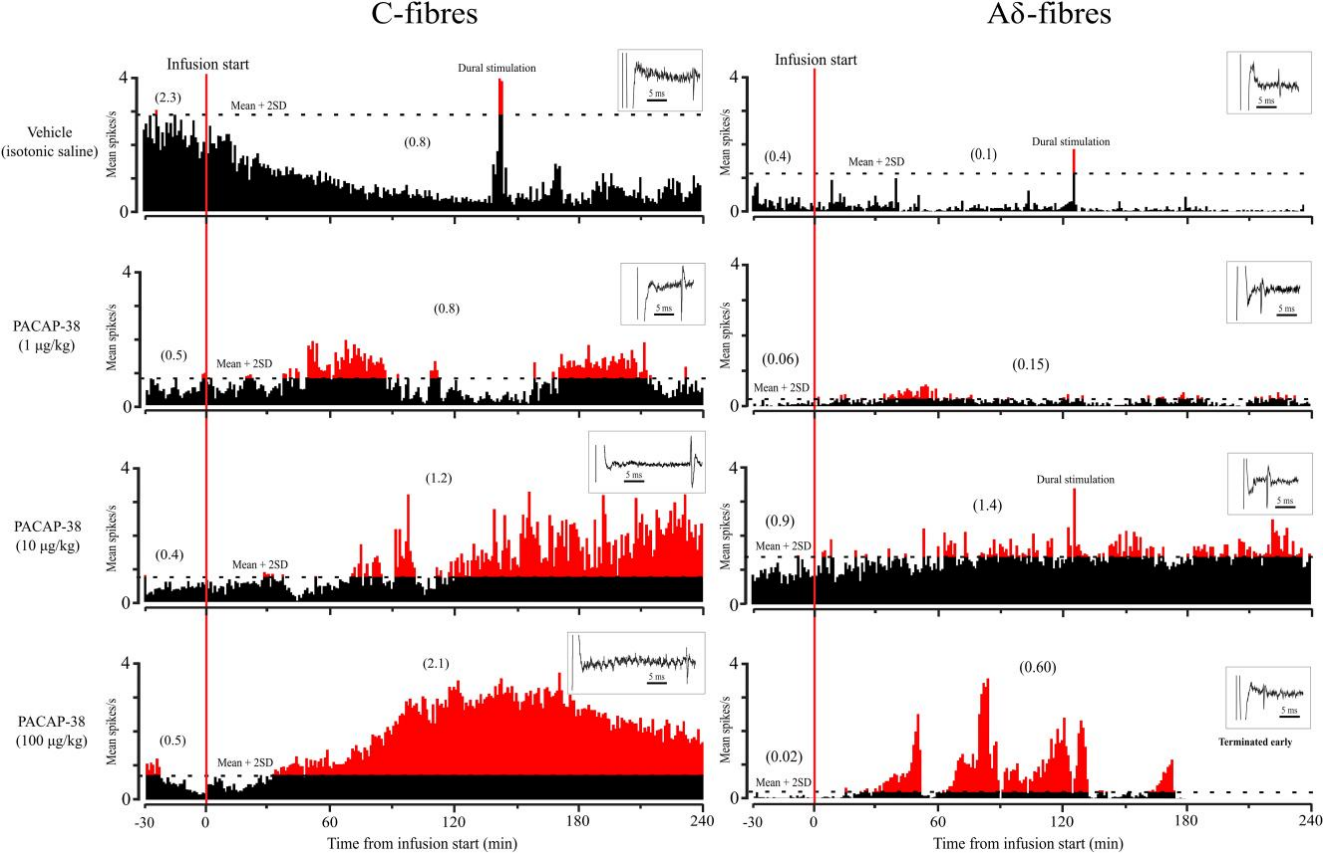
**Examples of neuronal firing rates after PACAP-38 and vehicle.** Firing rates of C- (*left*) and Aδ-type (*right*) meningeal nociceptors before and after infusion of vehicle (isotonic saline) or PACAP-38 at doses of 1 µg/kg, 10 µg/kg and 100 µg/kg (from *top* to *bottom*). Parenthesized numbers indicate firing rate before and after the start of infusion. SD = standard deviation.

Single-unit recordings were obtained from 25 Aδ (19 in male, 6 in female) and 28 C-fibre (19 in male, 9 in female) meningeal nociceptors within the trigeminal ganglion, identified through their response to electrical and mechanical stimulation of the dura covering the ipsilateral transverse sinus. We tested the effects of a 20-min intracarotid PACAP-38 infusion at various concentrations on a total of 43 neurons. Specifically, 10 µg/ml/kg PACAP-38 (human-equivalent dose) was tested in 30 neurons (13 Aδ, 17 C), 1 µg/ml/kg PACAP-38 in 9 neurons (4 Aδ, 5 C) and 100 µg/ml/kg PACAP-38 in 4 neurons (2 Aδ, 2 C). In addition, vehicle effects were assessed in 10 neurons (6 Aδ, 4 C). See Fig. 2 for examples of neuronal data at each dose.

### Baseline firing rates

The overall median baseline firing rate was 0.52 (IQR 0.19–1.09) spikes/s. Furthermore, the median baseline firing rates did not differ between Aδ-fibres (0.49; IQR 0.18–0.96 spikes/s) and C-fibres (0.50; IQR 0.16–0.70 spikes/s; *P* = 0.30). Similar median baseline rates were also seen in neurons that were activated by PACAP-38 (0.45; IQR 0.19–1.00 spikes/s), compared to those not activated (0.87; IQR 0.32–1.19 spikes/s; *P* = 0.08; Table 1).

**Table 1:** Summary of results for PACAP-38, 10 μg/ml (human equivalent dose)

	Aδ-fibres	C-fibres	*P*-value (Aδ versus C)
Number	13	17	–
Female, *n* (%)	4 (30.8)	6 (35.3)	1
Baseline firing rate, Hz; median [IQR]	0.49 [0.18–0.96]	0.97 [0.45–1.27]	0.303
Latency, in ms; median [IQR]	5.7 [4.5–7.5]	13.5 [11.5–16.9]	NA
Activated, *n* (%)	8 (61.5)	7 (41.2)	0.462
Time to onset, in min; median [IQR]^a^	103 [53.5–179.5]	91 [56–171.5]	1
Duration, in min; median [IQR]^a,b^	60.5 [34.25–90.5]	55 [39–90]	0.959

IQR = interquartile range, NA = not applicable.

^a^For activated neurons.

^b^Number of bins above baseline mean + 2 standard deviations.

### Meningeal nociceptors activation by PACAP-38

#### Activation rate

**Figure 3:**
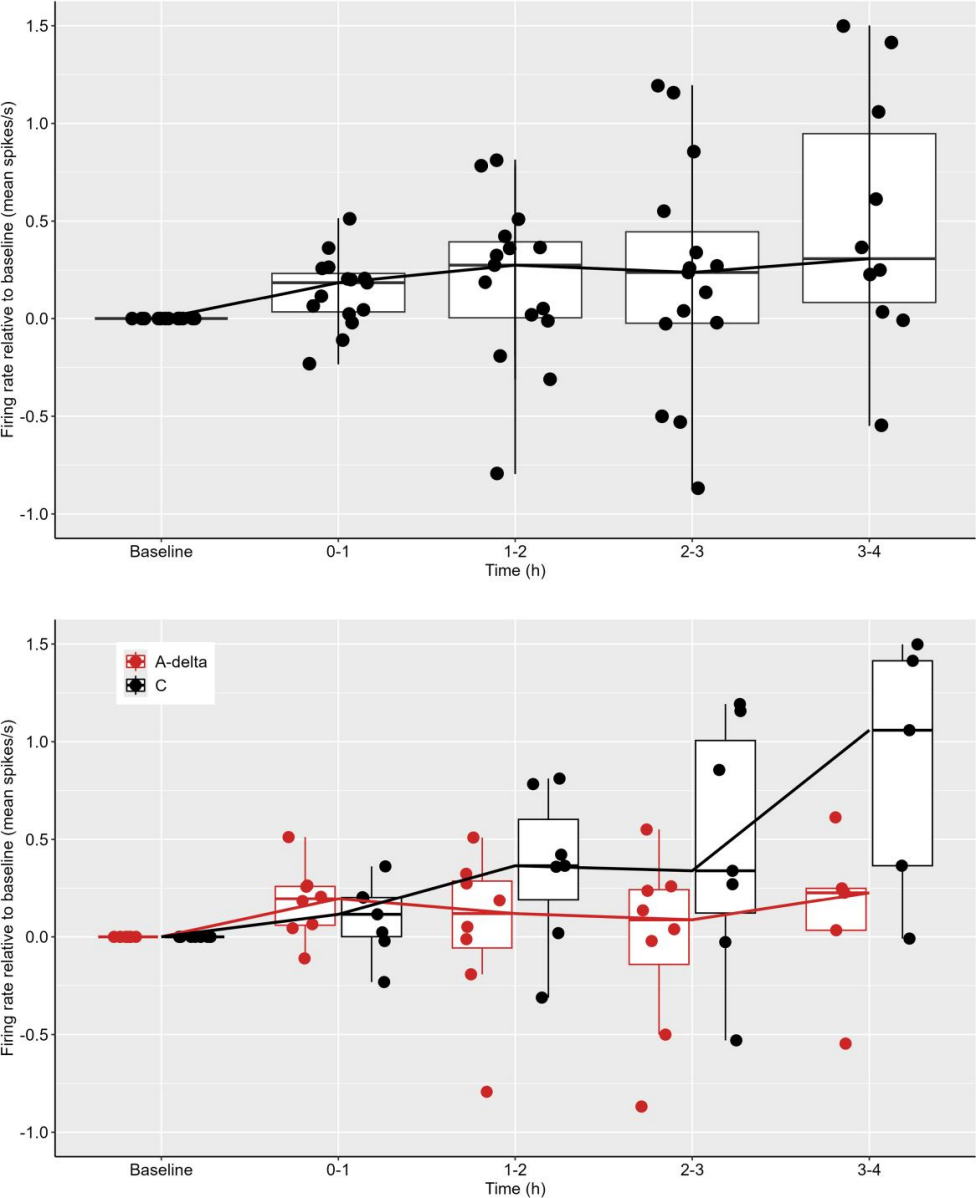
**Neuronal firing rates after PACAP-38 relative to baseline.** Firing rate relative to baseline as mean spikes/s in neurons activated by PACAP-38, 10 µg/kg. *Top*: All neurons. *Bottom*: Separated according to fibre type.

At the human-equivalent dose of 10 µg/ml/kg, intracarotid PACAP-38 infusion activated 15 of 30 (50.0%) neurons (Fig. 3, top). In comparison, none of the 10 neurons (6 Aδ, 4 C) studied after vehicle exhibited activation (*P* < 0.01). At the same dose (10 µg/ml/kg), PACAP activated 8 of 13 (61.5%) of the Aδ-fibres and 7 of 17 (41.2%) of the C-fibres (Fig. 3, bottom). The activation rate did not significantly differ between these two classes of meningeal nociceptors (*P* = 0.46). Comparable activation rates were also observed in male (10 of 20, 50.0%) and female (5 of 10, 50%) rats (*P* = 1.00). Therefore, we pooled data from male and female rats in all subsequent analyses.

#### Response latency

The median time to onset of activation was 97 (IQR 55–175.8) min, with no significant differences between Aδ-fibres (103; IQR 53.5–179.5 min) and C-fibres (91; IQR 56–171.5 min; *P* = 1.00).

#### Response magnitude

**Figure 4:**
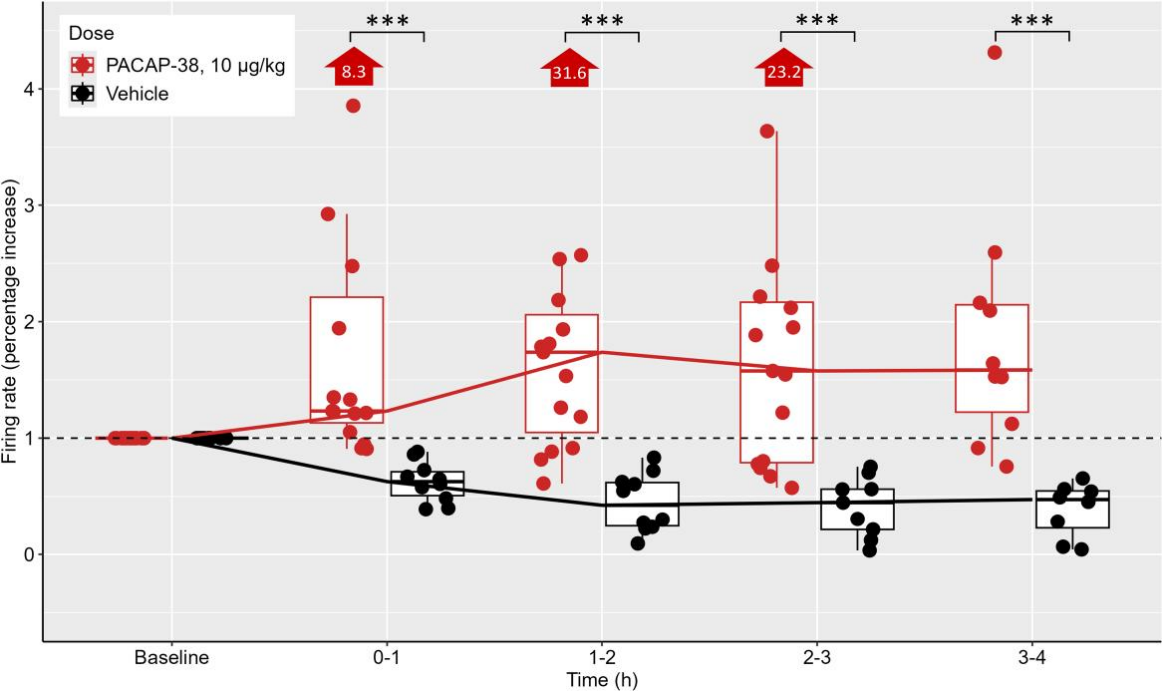
**Neuronal firing rates after PACAP-38 and vehicle relative to baseline.** Change in firing rate relative to baseline (fold increase or decrease) in neurons activated by PACAP-38, 10 µg/kg, compared to vehicle; displayed for all neurons. Note three data points above the edge of the plot for PACAP-38, 10 µg/kg at 0–1 (8.3-fold increase), 1–2 (31.6-fold increase) and 2–3 h (23.2-fold increase). ***Significant at *P* < 0.001.

At 10 µg/ml/kg, PACAP-38 elicited a 130% increase in firing rate among all activated neurons, rising from 0.70 (IQR 0.20–1.78) spikes/s at baseline to 1.63 (IQR 0.48–2.01) spikes/s (*P* = 0.037). The firing rate in activated Aδ-fibres increased by 430%, from 0.45 (IQR 0.23–0.97) spikes/s at baseline to 1.95 (IQR 0.20–2.04) spikes/s (*P* = 0.13; Fig. 3). In activated C-fibres, firing rate rose by 60%, from 0.96 (IQR 0.20–2.04) spikes/s at baseline to 1.59 (IQR 0.42–1.70) spikes/s (*P* = 0.44; Fig. 3). The percentage increase in firing rate after 10 µg/ml PACAP-38 exceeded vehicle at each observational hour (Hour 1, *P* < 0.01; Hour 2, *P* < 0.01; Hour 3, *P* < 0.01; Hour 4, *P* = 0.04; Supplementary Fig. 1). The effect was even more pronounced among neurons activated by PACAP-38 (Hour 1, *P* < 0.01; Hour 2, *P* < 0.01; Hour 3, *P* < 0.01; Hour 4, *P* < 0.01; Fig. 4 and Supplementary Fig. 2). The mean duration of PACAP-38–induced neuronal activation was 73.5 min (SD 46.7; range 13–191). Notably, in 8 of 15 activated neurons, firing had not yet ceased when the experiments were ended. The mean activation duration did not differ significantly between Aδ-fibres (66.9 min; SD 40.7; three ended prematurely) and C-fibres (81.0 min; SD 51.8; five ended prematurely; *P* = 0.68).

#### Activation rate at lower and higher concentrations

At 1 µg/ml/kg, PACAP-38 infusion activated three of nine (33%) neurons, while at 100 µg/ml/kg, it activated all four tested neurons (100%; Supplementary Tables 1 and 2).

### Meningeal nociceptors sensitization by PACAP-38

#### Threshold

**Figure 5:**
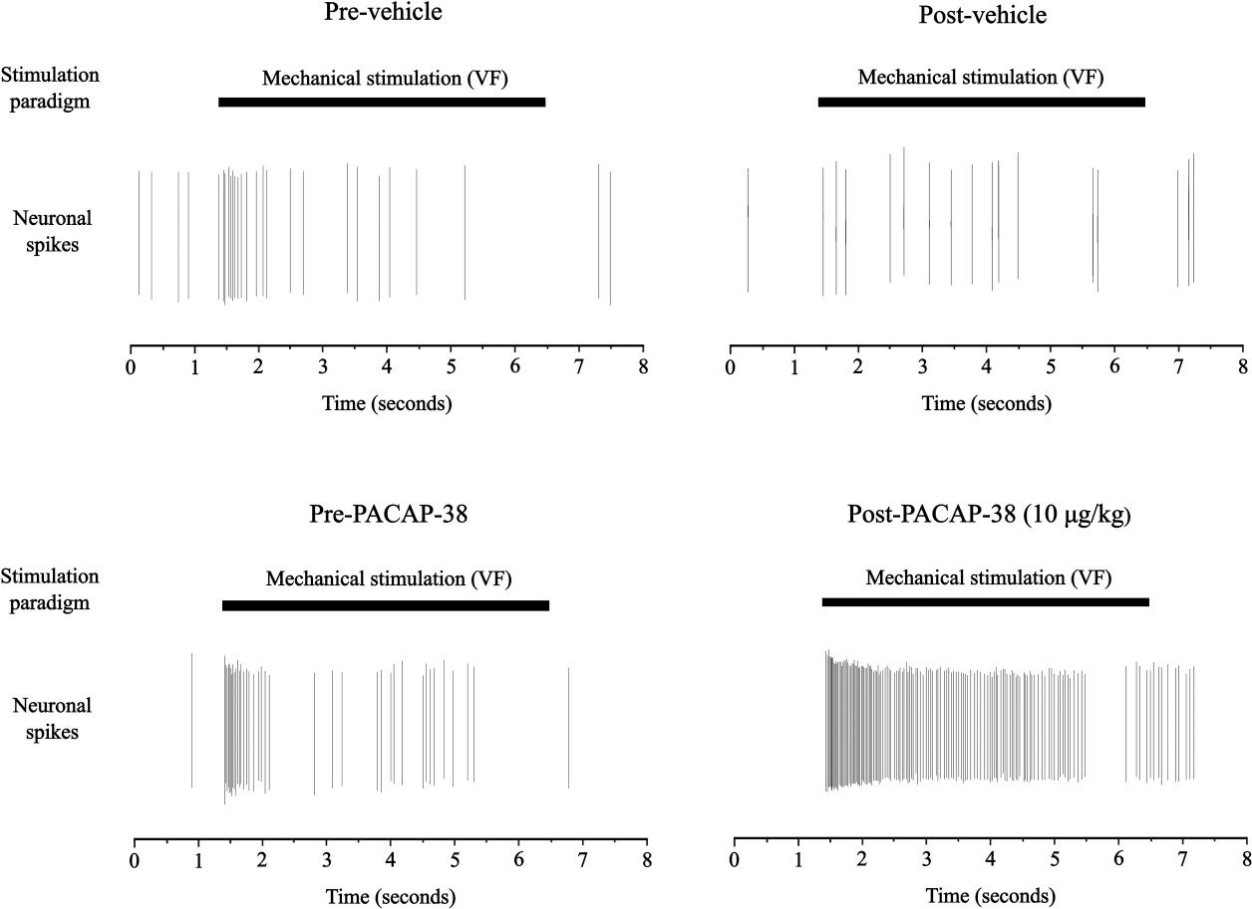
**Example of sensitization after PACAP-38.** Example of sensitization displaying neuronal spikes fired during a 5-s mechanical dural stimulation (2 g von Frey filament) before and after administration of either vehicle (isotonic saline) or PACAP-38 (10 µg/ml). *Top left*: Neuronal spikes during mechanical stimulation before administration of vehicle in a C fibre. *Top right*: Neuronal spikes during mechanical stimulation after administration of vehicle in the same neuron. *Bottom left*: Neuronal spikes during mechanical stimulation before administration of PACAP-38 in an Aδ fibre. *Bottom right*: Neuronal spikes during mechanical stimulation after administration of PACAP-38 in the same neuron. VF = von Frey.

**Figure 6:**
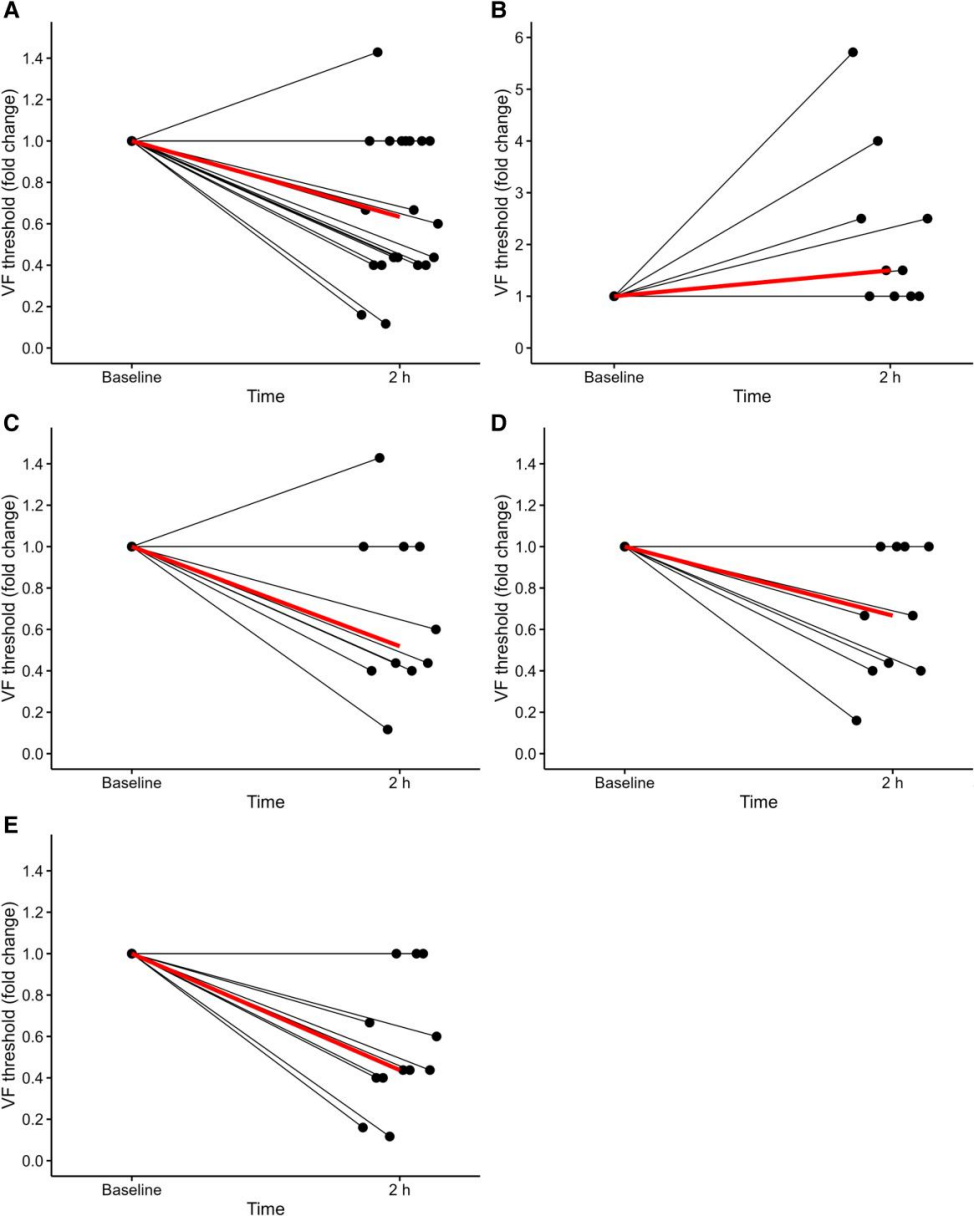
**Sensitization after PACAP-38.** Sensitization and change in mechanical threshold between baseline and 2 h after PACAP-38, 10 µg/ml, for (**A**) all neurons, and (**B**) vehicle (isotonic saline), (**C**) Aδ-fibres, (**D**) C-fibres, and (**E**) neurons activated by PACAP-38. VF = von Frey.

Two hours after a 10 µg/ml/kg PACAP-38 infusion, 12 of 20 (60%) meningeal nociceptors demonstrated decreased mechanical thresholds, compared with 0 of 10 (0%) following vehicle (*P* < 0.01). Figure 5 provides representative examples of this sensitization, and Fig. 6 provides overviews. The proportion of sensitized neurons was identical in Aδ-fibres (60%) and C-fibres (60%) (*P* = 1.00; Table 2). Sensitization was observed in a similar proportion of nociceptors from male (7 of 10, 70%) and female (5 of 10, 50%) rats (*P* = 0.65).

**Table 2:** Mechanical threshold (von Frey filament) for activation and magnitude of response to mechanical stimulation for neurons examined with PACAP-38, 10 μg/ml

	Aδ-fibres	C-fibres	*P*-value (Aδ versus C)
Number	10	10	–
Female, *n* (%)	4 (40.0%)	6 (60.0%)	0.656
Number sensitized to mechanical stimulation, *n* (%)	6 (60)	6 (60)	1
Threshold at baseline, g [IQR]	0.4 [0.16–0.9]	0.5 [0.16–0.6]	0.939
Threshold at 2 h, g [IQR]	0.12 [0.07–0.4]	0.16 [0.16–0.4]	0.536

IQR = interquartile range.

#### Magnitude

The magnitude of mechanical threshold reduction was assessed in each fibre type. Among the sensitized Aδ-fibres, the threshold decreased by 76%, from 0.5 (IQR 0.22–0.9) g at baseline to 0.12 (IQR 0.07–0.34) g at 2 h post-PACAP-38 infusion (*P* = 0.04). Sensitized C-fibres exhibited a 53% reduction, from 0.6 (IQR 0.45–0.90) g at baseline to 0.28 (IQR 0.16–0.4) g (*P* = 0.04).

### Effects of dural lidocaine

To determine the role of dural receptive fields in ongoing activation and sensitization of meningeal nociceptors that exhibited increased firing for several hours after PACAP-38 (10 µg/ml/kg) infusion, lidocaine was applied topically to the receptive field. A dose of 20 mg/ml lidocaine was applied to the exposed dura mater above the transverse sinus of neurons, and the firing rate was assessed for 15–20 min. In two experiments (1 Aδ, 1 C), lidocaine abolished firing completely, while in three experiments (1 Aδ, 2 C), firing decreased by 40%–55% (Supplementary Fig. 3).

### Physiological effects of PACAP-38

The human equivalent dose of PACAP-38 (10 µg/ml/kg) significantly increased heart rate relative to baseline at 10, 20 and 30 min after start of infusion, when compared to vehicle (10 min: *P <* 0.01, 20 min: *P* < 0.01, 30 min: *P* = 0.04; Supplementary Table 3 and Supplementary Fig. 4).

## Discussion

Using single-unit electrophysiological recordings, we demonstrated that intracarotid administration of PACAP-38 at 10 µg/kg (the human-equivalent dose) activates and sensitizes Aδ- and C-fibre meningeal nociceptors in both male and female rats. This dose elicited activation and sensitization in about half of all tested nociceptors. Both classes of nociceptors exhibited comparable response latencies (about 90 min) and duration (often at least 70 min), although the response magnitude was slightly greater in Aδ-fibres. Application of lidocaine onto the dura mater several hours after PACAP-38 infusion attenuated or completely abolished discharge activity of both Aδ- and C-fibres. Based on these findings, we conclude that PACAP-38 induces migraine headache in people with migraine by activating and sensitizing meningeal nociceptors. This conclusion questions previous interpretations that systemic PACAP administration does not activate the peripheral component of trigeminovascular pain pathways.^16^

### Clinical correlates

Our findings provide a mechanistic basis for clinical studies showing that PACAP-38 induces headache, migraine and cluster headache attacks in both men and women.^14,17,18^ The delayed onset of migraine in patients (range 2–11 h)^14^ can be explained by the 90 min delayed activation of the nociceptors. The aggravation of headache by physical activity and throbbing character of pain can be explained by the sensitization of meningeal nociceptors to mechanical stimulation and arterial pulsation—even if throbbing is not univocally synchronized with pulsation.^19^ And the duration of the induced headache can be explained by the persistent activation (range 13–191 min, often persisting through the end of the recording) of the nociceptors. Since PACAP-38 is quickly eliminated from the body (half-life of 3.5 min in humans^20^ and 0.8 min in rats^21^) and the infusion lasted 20 min, it is reasonable to propose that PACAP-38 does not directly depolarize the primary afferents, but rather initiates signalling cascades that eventually render the nociceptors more excitable and closer to activation. The exact molecular underpinnings of such delayed effects remain uncertain, but the findings are consistent with delayed nocifensive behaviour in animal models.^22,23^ This delayed effect might be suggestive of indirect mechanisms of activation, with some lines of evidence indicating that PACAP-38 promotes sustained mast cell degranulation that may prolong vasodilation and contribute to gradual sensitization of nociceptors.^22,24,25^ Another option is that PACAP-38 acts directly on the meningeal nociceptors to initiate intracellular processes that continuously excite nociceptors towards activation.

### PACAP-38 mechanisms of action

The presence of PACAP-38 and its receptors on dural nociceptors and non-neuronal cells^26-28^ give rise to three possible mechanistic explanations for its ability to trigger headache: (i) direct effects on meningeal nociceptors; (ii) indirect effects on meningeal nociceptors, through dilation of cranial vessels and the release of potassium from vascular smooth muscle cells; or (iii) indirect effects, through mast cell degranulation and ensuing effects of chemical mediators on nociceptors, directly or indirectly through the vasculature.

#### Neuronal effect

PACAP is thought to be released in the dura from activated parasympathetic and sensory nerve endings.^6-8^ The mechanisms by which PACAP-38 excites meningeal nociceptors are not fully delineated. Although all three PACAP receptors (VPAC_1_, VPAC_2_ and PAC_1_) are expressed in primary afferents of the rat and human trigeminal ganglion,^29^ their expression on Aδ relative to C-fibres remains unknown. Binding of PACAP-38 to its G protein-coupled receptors upregulates cAMP, initiating a series of intracellular signalling pathways involving phosphorylation,^30,31^ upregulation of ion channels and receptors,^32,33^ long-term potentiation,^34^ neurotransmitter synthesis and vesicular transport,^35,36^ synaptic plasticity,^37^ neuronal hyperexcitability and pain chronification.^34^ Given the complex extracellular-intracellular process by which PACAP-38 can affect neuronal excitability, it is more likely to act as a primer than a stimulator—which might account for the delayed activation of the nociceptors. Regardless of the exact mechanisms, PACAP-38 activated both the unmyelinated C- and the thinly myelinated Aδ-fibres with a similar latency—suggesting either similar indirect effects or that PACAP receptors are found on both the C- and Aδ-fibres.^29^

#### Vascular effect

Another option is that the activation of meningeal nociceptors by PACAP-38 is secondary to its effect on dural vasculature. The VPAC_1_, VPAC_2_ and PAC_1_ receptors are present on dural arteries in humans and rat,^10,12^ where they upregulate intracellular cAMP to activate protein kinases. These phosphorylate and open ATP-sensitive as well as large-conductance Ca^2+^-activated potassium channels,^38^ which results in dilation of meningeal arteries^25^—potentially setting the stage for eventual activation of the nociceptors. In this regard, we cannot rule out that the mechanical stretch from arterial dilatation might suffice to activate the sensitized nociceptors. Importantly, another possibility is that the efflux of potassium from vascular smooth muscle cells might generate a chemical gradient that could also play a role in the enhanced excitability of the meningeal nociceptors.^39^

#### Mast cell effects

Interacting with MrgB3 receptors in rats and MrgX2 receptors in humans, PACAP-38 has been shown to degranulate dural mast cells.^13^  ^,24^ Mast cell degranulation releases a host of mediators, such as histamine and prostaglandin I_2_, which can activate meningeal nociceptors^40^ and provoke headache in healthy volunteers and migraine attacks in people with migraine.^41-43^ These same mast cell mediators also dilate meningeal vessels through cAMP-dependent mechanisms.^44,45^ A potential role of histamine is supported by findings that the H1 receptor antagonist clemastine descriptively reduced PACAP-38 induced migraine from 45% to 25% in one study.^46^

### Prior studies

Our findings challenge previous conclusions regarding the role of PACAP-38 within the trigeminovascular system. Akerman and colleagues^16^ previously demonstrated that intravenous administration of PACAP-38 induced delayed sensitization of second order trigeminovascular neurons in the spinal trigeminal nucleus (SpV). Their results also revealed that intracerebroventricular, but not intravenous administration, of PAC1 and VPAC1 receptor antagonists attenuated the central neuronal responses to electrical stimulation of the dura. These findings led them to propose that PACAP-38 triggers migraine headache through PAC1 receptor activation in the SpV rather than by influencing meningeal nociceptors. Hence, they concluded that migraine headache results from direct activation and sensitization of central trigeminocervical neurons.

However, evidence that peripheral administration of PACAP-38 triggers distinct activation and sensitization of both C- and Aδ-meningeal nociceptors with a delay that closely resembles the human experience, and that topical administration of lidocaine can abolish or attenuate the PACAP-38 induced firing of the nociceptors, suggests that PACAP-38 can trigger migraine headache by activating meningeal nociceptors. This conclusion offers a far more convincing explanation for PACAP-38’s ability to trigger a migraine-like headache, since the individual central trigeminovascular neurons process nociceptive information from both the dura as well as periorbital skin, cornea, pericranial muscles and other trigeminal structures. One would therefore expect them to produce generalized trigeminal pain rather than classical migraine headache. In contrast, the meningeal nociceptors retain innervation more specifically relevant to headache, and we therefore propose that the most plausible way to explain PACAP-38 ability to trigger a migraine is through its ability to activate peripheral rather than central trigeminovascular neurons.

Our conclusion that the mechanism by which PACAP-38 triggers headache involves its effect on meningeal nociceptors does not contradict the data in Akerman’s study,^16^ but suggests that the conclusions were premature for a number of reasons. First, Akerman’s study examined the effect of PAC1 receptor antagonism on unevoked firing rates and dural responses, instead of investigating whether PAC1 antagonisms inhibited PACAP’s ability to activate the second order neurons. The latter would seem more relevant to PACAP’s mechanism of action. Second, we note that the time course of Akerman’s study was vastly different compared to ours. Effects of antagonists were investigated for only 45 min—but it took 90 min for PACAP to activate meningeal nociceptor in the current study, and to increase the firing rate of second order neurons in Akerman’s study. Third, peripheral effects might be mediated by other receptors, such as MrgB3, which were not investigated in the study. Overall, it seems likely that activation of second-order neurons was secondary to activation of meningeal nociceptors. This is consistent with evidence for limited BBB penetration of PACAP-38, including absence of middle cerebral artery dilation (protected by the blood–CSF barrier) and the lack of central side effects to PACAP-38 infusion and anti-PACAP monoclonal antibodies.^15,47^ Moreover, the 4534 dalton size of PACAP-38 exceeds the generally accepted 400–600 dalton limit for passing the BBB. Nevertheless, some reports suggest small amounts of PACAP-38 might cross the BBB, but are rapidly removed by efflux pumps.^48^

Beyond the specific scope of Akerman et al.’s study,^16^ it is however therapeutically relevant to note that PACAP receptors are distributed across several brain areas such as the lateral geniculate nucleus, hypothalamus, thalamus, frontal cortex, cerebellum, hippocampus and multiple nuclei of the brainstem.^49,50^ This widespread distribution calls attention to the possibility that blocking PACAP signalling in the brain might cause side effects related to disruption of vision, cognition, circadian rhythm, behaviour, mood regulation and neuroprotection. Targeting PACAP signalling outside the BBB may therefore provide an overall safer and more efficacious approach for novel treatments. Even so, it is important to consider that some, albeit limited, PACAP might potentially cross the BBB, as well as the possibility of systemic side effects. Most recently, results from a recent phase II clinical trial have however suggested good tolerability and safety of an anti-PACAP antibody, which, due to its large size, is unlikely to cross the BBB. Likewise, one would expect similar systemic side effects for brain penetrant versus non-penetrant inhibitors.

### PACAP-38 versus CGRP

Given the success of treatments targeting CGRP for migraine, it is reasonable to note a number of similarities with PACAP-38. Like PACAP-38, CGRP is a neuropeptide capable of inducing mild headache in healthy controls, migraine attacks in individuals with migraine, migraine-like headache in individuals with persistent post-traumatic headache, and cluster headache attacks in individuals with cluster headache.^51-53^ Likewise, both are vasodilatory peptides expressed within the dura and the trigeminal system.

However, there are also key differences. Whereas CGRP is expressed in trigeminal sensory afferents, PACAP-38 has relatively low trigeminal expression and is considered to be released mainly from parasympathetic efferents. The canonical CGRP receptor is seemingly only expressed on Aδ nerve fibres, especially considering that CGRP receptor antagonists and antibodies preferentially inhibit Aδ-fibres. In contrast, our results suggest that PACAP receptors might be expressed on some of both C- and Aδ-fibres or that PACAP-38 acts indirectly. There are still limited data for the expression of PACAP receptors within meningeal nociceptors, and further determining the distribution of dural PACAP receptors will be necessary to elucidate whether the activation and sensitization of Aδ- or C-type fibres occur through direct or indirect mechanisms. Nevertheless, given evidence for the presence of CGRP receptors on Aδ- but not C-fibres, it is possible that the mechanisms of action by which CGRP triggers migraine headache differ from PACAP-38. If correct, this finding can give rise to future attempts to combine drugs that inhibit CGRP signalling with drugs that inhibit PACAP signalling in the treatment of migraine. Likewise, in a behavioural study, PACAP-38 induced periorbital hypersensitivity independent of the CGRP receptor, as evaluated in RAMP1 knockout mice.^54^ These differences are intriguing in that they suggest that combining treatment targeting PACAP-38 and CGRP might yield improved efficacy compared to monotherapy. In addition, they also provide mechanistic insights that targeting PACAP may be beneficial for nonresponders to anti-CGRP treatments.

### Outstanding questions

Yet to be determined are the specific molecular mechanisms whereby PACAP activates meningeal nociceptors. A crucial goal for future studies would be to investigate the contribution of specific receptors to nociceptor activation and sensitization, by comprehensively examining antagonism of the three PACAP receptors as well as the mast cell receptor MrgB3/MrgX2. Moreover, further exploration of intracellular signalling pathways downstream of PACAP receptors, such as those involving cAMP/PKA, could provide important insights into especially sustained activity within meningeal nociceptors. Finally, we note that interspecies differences might exist in the pharmacology and distribution of PACAP-38 and its receptors. For example, some evidence suggests VPAC2 might have a more prominent role in meningeal dilation in humans, whereas VPAC1 might also have a role in rats.^55^ In contrast, PACAP appears to potently degranulate both rat and human mast cells, despite different MrgB3/MrgX2 isoforms.^24^ Nevertheless, the close alignment of our models and dosages with those used in humans—along with the consistency of our findings with observations that PACAP infusion induces headaches and migraines in patients, triggers nocifensive behaviour in animal models and causes meningeal dilation in both—strongly suggests that our results account for observations in patients.

## Conclusion

In summary, our study provides compelling evidence that PACAP-38 activated and sensitized meningeal nociceptors. The activation was sustained, robust, dose-dependent and susceptible to dural lidocaine blockade. There were no clear differences between Aδ- and C-fibres or male and female animals in terms of activation or sensitization. Our results imply that PACAP-38’s ability to induce headache is mediated primarily through its ability to sensitize and activate meningeal nociceptors outside the BBB. This provides further evidence supporting the development of novel migraine therapies that target peripheral structures.

## Supplementary Material

awaf284_Supplementary_Data

## Data Availability

Data will be made available from the corresponding author upon reasonable request from a qualified investigator.
