# Supplementary material for: Activation and sensitization of meningeal nociceptors by PACAP-38: implications for migraine headache
Source: Brain. 2025 Aug 6;149(3):897–907. doi: 10.1093/brain/awaf284 (PMC13017544; doi:10.1093/brain/awaf284)
Supplement: awaf284_Supplementary_Data [file awaf284_supplementary_data.docx]

**Supplementary Tables**

**Supplementary Table 1.** Summary of results for PACAP-38, 1 μg/ml

|  | **Aδ-fibers** | **C-fibers** | ***P*-value**  **(Aδ vs C)** |
| --- | --- | --- | --- |
| Number | 4 | 5 | - |
| Latency, ms, median [IQR] | 4.7 [4.7-4.7] | 15.25 [13.63-16.88] | 0.667 |
| Activated, n (%) | 1 (25.0) | 2 (40.0) | 1 |
| Time to onset, minutes, median [IQR]* | 41 [41-41] | 24.5 [12.25-36.75] | 0.667 |
| Duration, minutes, median [IQR]†* | 60.5 [34.25-90.5] | 55 [39-90] | 0.667 |

*For activated neurons.
†Number of bins above baseline mean + 2 standard deviations.

**Supplementary Table 2.** Summary of results for PACAP-38, 100 μg/ml

|  | **Aδ-fibers** | **C-fibers** |
| --- | --- | --- |
| Number | 2 | 2 |
| Latency, ms (for each neuron) | 6.5 and 7.5 | 20 and 28 |
| Activated, n (%) | 2 (100.0) | 2 (100.0) |
| Time to onset, minutes, median [IQR]* | 23 [19.75-24.74] | 17.5 [15.25-19.75] |
| Duration, minutes, median [IQR]†* | 117 [104.5-129.5] | 154 [122.5-185.5] |

*For activated neurons.
†Number of bins above baseline mean + 2 standard deviations.

**Supplementary Table 3.** Heart rate change relative to baseline for PACAP-38, 10 μg/ml (human equivalent dose), compared to vehicle.

| **Time point (min)** | **PACAP-38, 10 μg/kg,**  **mean (SD)** | **Vehicle,**  **mean (SD)** | ***P*-value** |
| --- | --- | --- | --- |
| 10 | 3.2 (5.6) | -1.2 (2.4) | 0.002* |
| 20 | 3.4 (8.4) | -2.0 (2.9) | 0.002* |
| 30 | 0.8 (8.0) | -3.3 (3.3) | 0.036* |
| 40 | -0.8 (6.9) | -1.8 (4.9) | 0.598 |
| 50 | -2.0 (7.9) | -3.4 (3.8) | 0.488 |
| 60 | -2.1 (10.8) | -3.7 (4.7) | 0.525 |

*Significant at *P* ≤ 0.05.

**Supplementary Figures**

**
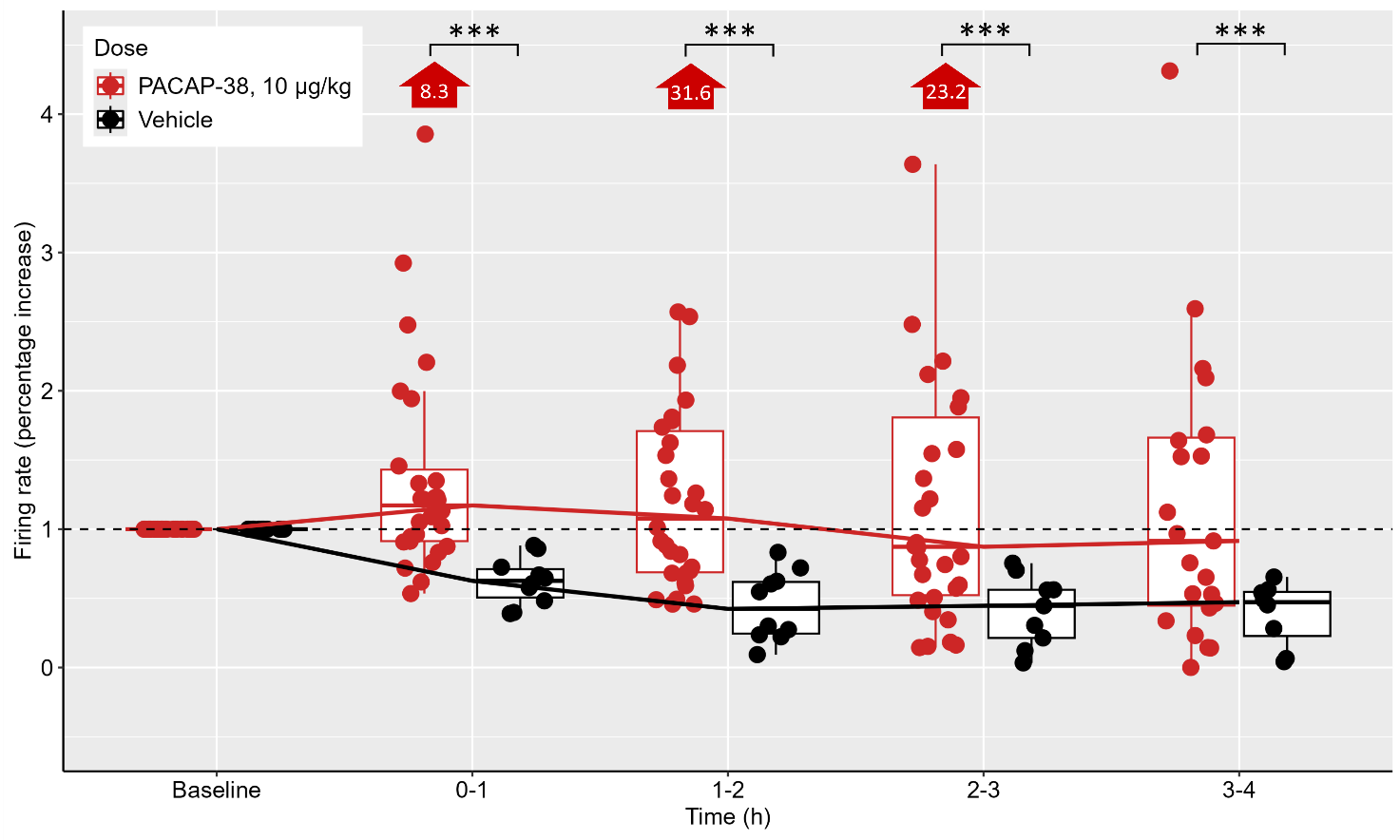
Supplementary Figure 1.** Change in firing rate (fold increase or decrease relative to baseline) in all neurons investigated with PACAP-38, 10 μg/kg (red symbols) compared to vehicle (black symbols). Left: displayed for all neurons. Note three datapoints above the edge of the plot for PACAP-38, 10 μg/kg at 0-1 (8.3 fold increase), 1-2 (31.6 fold increase), and 2-3 hours (23.2 fold increase).

***Significant at *P* < 0.001, **Significant at *P* < 0.01, *Significant at *P* < 0.05.


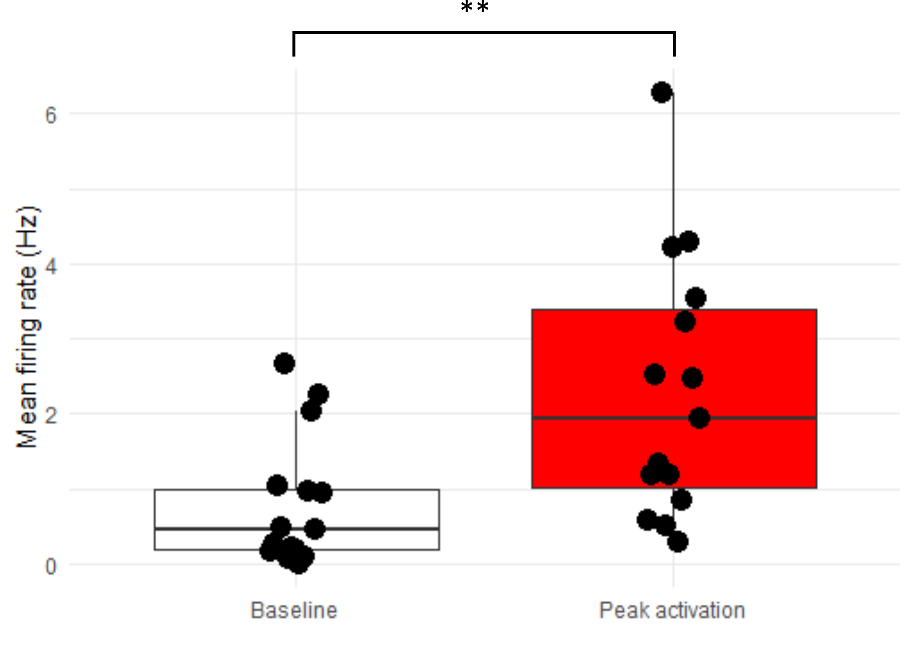


**Supplementary Figure 2.** Increase in firing rate in neurons activated by PACAP-38, 10 μg/ml, during activation, compared to baseline. **significant at *P* < 0.01.


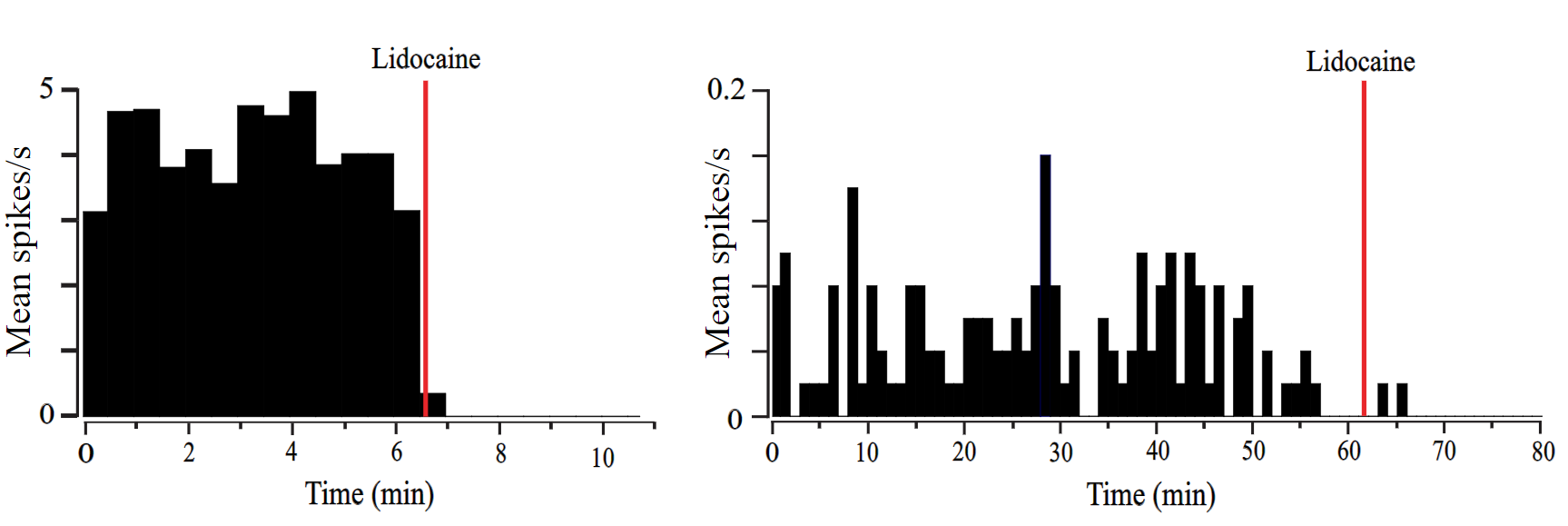


**Supplementary Figure 3.** Examples of dural lidocaine (2%) inhibiting activation after PACAP-38, 10 μg/ml. Left panel: Aδ fiber. Right panel: C-fiber.

**
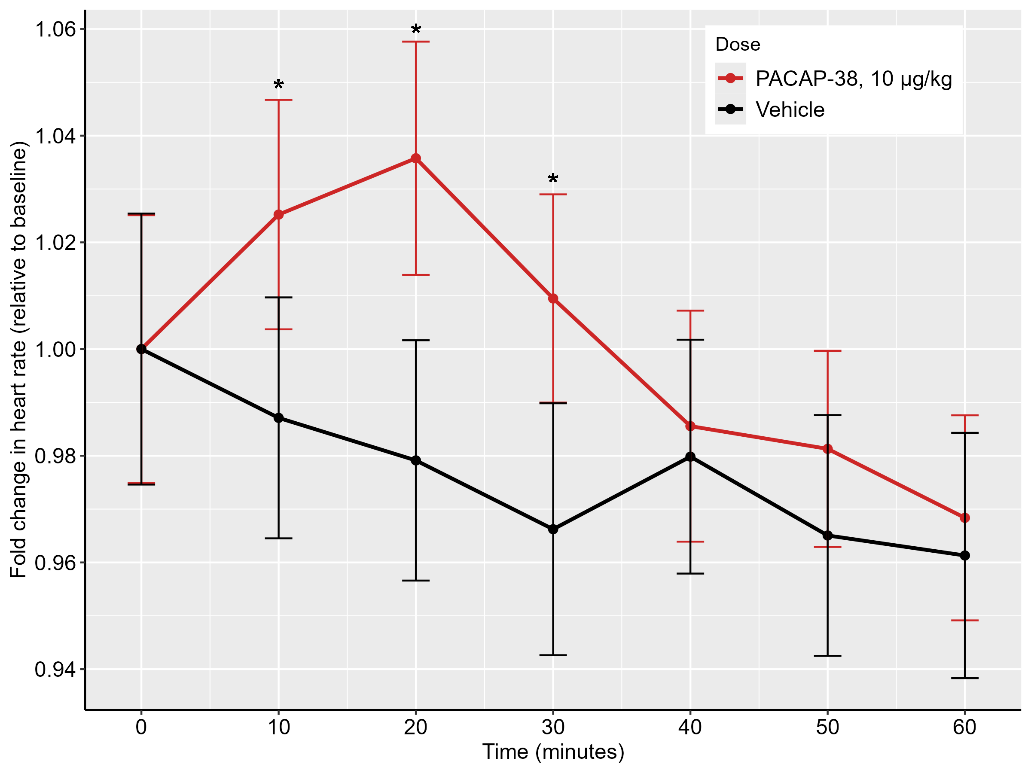
**

**Supplementary Figure 4.** Mean change in heart rate from baseline after PACAP 10 µg/kg and vehicle (isotonic saline). Error bars are standard errors of the mean. *Significant at *P* ≤ 0.05.
